# Efficacy, safety, and economic assessment of hominis placental pharmacopuncture for chronic temporomandibular disorder: a protocol for a multicentre randomised controlled trial

**DOI:** 10.1186/s13063-020-04442-8

**Published:** 2020-06-15

**Authors:** Jongho Kim, Kyoung Sun Park, Yoon Jae Lee, Koh-Woon Kim, Jae-Heung Cho, In-Hyuk Ha

**Affiliations:** 1 Jaseng Hospital of Korean Medicine, 536 Gangnam-daero, Gangnamgu, Seoul, Republic of Korea; 2 Jaseng Spine and Joint Research Institute, Jaseng Medical Foundation, 3F JS Tower, 538 Gangnam-daero, Gangnam-gu, Seoul, Republic of Korea; 3 Kyung Hee University Hospital at Gangdong, 892 Dongnam-ro, Gangdong-gu, Seoul, Republic of Korea; 4 Kyung Hee University, 26 Kyungheedae-ro, Dongdaemun-gu, Seoul, Republic of Korea

**Keywords:** Pharmacopuncture, Hominis placental pharmacopuncture, Temporomandibular disorder, Randomised controlled trial, Protocol

## Abstract

**Background:**

Temporomandibular disorder (TMD) is a condition encompassing clinical symptoms of the temporomandibular joint, masseter muscle, and surrounding structures. Hominis placental pharmacopuncture (HPP), consisting of human placental extract, has been reported as effective for treating chronic musculoskeletal disorders, but a lack of well-designed randomised controlled trial s (RCTs) mean there is insufficient evidence to prove the efficacy of HPP.

**Methods:**

This study is a two-arm parallel, assessor-blinded, multi-centre, randomised controlled trial. We will enrol 82 chronic TMD patients from rwo Korean Medicine hospitals in Axis 1, Group I according to RDC/TMD diagnostic criteria, and randomly allocate 41 patients each to an HPP group and a physical therapy (PT) group. Treatment will be administered in 10 rounds, after which there will be four follow-up visits 6, 9, 13, and 25 weeks from baseline. The primary end point is 6 weeks after baseline, and the primary outcome is the difference in Visual Analogue Scale (VAS) score for temporomandibular pain between baseline and week 6. Secondary outcomes will be Numeric Rating Scale (NRS) scores for temporomandibular pain and discomfort, temporomandibular joint range of motion, the Korean version of Beck’s Depression Index-II (K-BDI-II), Jaw Functional Limitation Scale (JFLS), Patient Global Impression of Change (PGIC) scores, and quality of life. Using data on adverse events and cost-effectiveness in the two groups, we will perform a safety assessment and a cost-effectiveness analysis (economic assessment).

**Discussion:**

This study will assess the efficacy and safety of HPP for chronic TMD compared with PT. This RCT will provide evidence for the efficacy, safety, and economics of HPP.

**Trial registration:**

clinicaTrials.gov (NCT04087005) / Clinical Research Information Service (CRIS) (KCT0004437) / IRB (JASENG 2017–09–002-002, KHNMCOH 2019–08-002) / Ministry of Food and Drug Safety (No. 31886).

## Background

Temporomandibular disorder (TMD) encompasses clinical symptoms of the temporomandibular joint (TMJ), masseter muscle, and surrounding structures. The most common symptoms of TMD are pain in the TMJ and masseter muscle, asymmetry, and restricted motion of the TMJ, and crepitus of the TMJ; these symptoms may also be accompanied by ear pain, tinnitus, dizziness, neck pain, and headaches. Symptom onset can be mild or acute and, like patients with chronic pain in other areas, continuous pain in the TMJ is often accompanied by physical, behavioural, and psychological symptoms [1]. Over 5% of the total population is estimated to have TMD, and although TMD affects people of various ages, its prevalence is especially high among 30–40year-olds, and is reported to be three times higher in females than in males [2]. According to data from the Health Insurance Review and Assessment Service (HIRA) [3], in South Korea a total of 390,000 patients received treatment for TMD in 2017, an increase of 24% from the previous 5 years.

Diagnostic tools for TMD include the American Academy of Orofacial Pain (AAOP) criteria and the International Headache Society classification [4], but the most widely used is the research diagnostic criteria for temporomandibular disorders (RDC/TMD) [5]. RDC/TMD is a multidimensional assessment that divides TMD into muscle and joint problems (Axis 1) and psychological problems (Axis 2) via a process of diagnosis and testing for symptoms including mouth opening, range of motion (ROM), pain, and discomfort. In Axis 1, TMD is classified into group I (myofascial pain), II (disc displacements) or III (other joint conditions), while Axis 2 examines the extent of pain, and the presence and extent of accompanying neuropsychological symptoms.

Conventional treatments for TMD include medication (oral, injection), physiotherapy, and surgery [1]. Drug therapy includes oral administration of non-steroidal anti-inflammatory drugs (NSAIDs), muscle relaxants, opioids, steroids, tricyclic antidepressants, selective serotonin reuptake inhibitors (SSRIs), anxiolytics, and antispasmodic drugs. However, the use of these drugs is restricted in patients with gastrointestinal adverse effects (AEs), or who are at risk of depression or drug dependence [6, 7]. In addition, local analgesics, corticosteroids, hyaluronic acid, ketamine, and botulinum toxin are administered by injection, but there is a lack of evidence supporting the efficacy of these treatments [8]. Physical therapy (PT) is widely used to treat TMD pain and dysfunction. Heat therapy and transcutaneous electrical nerve stimulation (TENS) are used to alleviate TMJ pain, whereas neck and jaw exercise programs, postural exercises, and manipulation of the jaw, neck, and face are used to improve TMJ ROM [6, 7, 9]. Surgical treatment includes arthroscopy, arthrocentesis, arthrotomy, and TMJ reconstruction [2]. However, AEs such as deafferentation pain mean it is only considered in patients who show no response after 3–6 months of conservative treatment and experience severe disruption to their daily lives, or in patients with structural, anatomical lesions. Other treatment methods include splinting, [10] cognitive behavioural therapy, counselling, and biofeedback [6, 7].

Acupuncture is the most widely used complementary and alternative medicine treatment for TMD [7]. It has been reported to be effective for musculoskeletal disease, especially pain and dysfunction of the TMJ [6, 11]). Pharmaco-acupuncture (PA) is a novel treatment combining acupuncture with herbal medicine, in which herbal medicine extracts are injected into acupoints [12, 13]. Hominis placental pharmacopuncture (HPP), which uses human placental extract as its core component, could be used in TMD treatment, but there have yet to be any well-designed, large-scale randomised control trials (RCTs) of HPP for TMD; the current level of evidence is low because most studies have thus far been small and allowed diverse concomitant treatments.

In this study, we aim to evaluate the efficacy, safety, and economics of HPP for chronic TMD. First, we will assess the comparative effectiveness and safety of HPP by comparing pain indices, functional indices, quality of life, and AEs between HPP and conventional treatment (PT). In addition, we will compare long-term cost-effectiveness between HPP and PT to assess whether HPP is a cost-effective treatment.

## Method/Design

The protocol for this study was compiled in accordance with SPIRIT 2013 Statement (Additional file 1) [14].

### Study design and setting

This study is a two-armed, parallel, multi-centre, randomised controlled study, and will be conducted at the Jaseng Hospital of Korean Medicine and Kyung Hee University Korean Medicine Hospital at Gangdong, located in South Korea. Prior to commencement, the study protocol has been approved by the institutional review board (IRB) at each centre (JASENG 2017–09–002-002, KHNMCOH 2019–08-002) and the Ministry of Food and Drug Safety (No. 31886). In addition, the study protocol has been registered at clinicaltrials.gov (NCT04087005) and Clinical Research Information Service (CRIS) (KCT0004437), and the state of the research will be continually updated. Information about the research centres and the researchers can be found at the trial registration site.

**Fig. 1:**
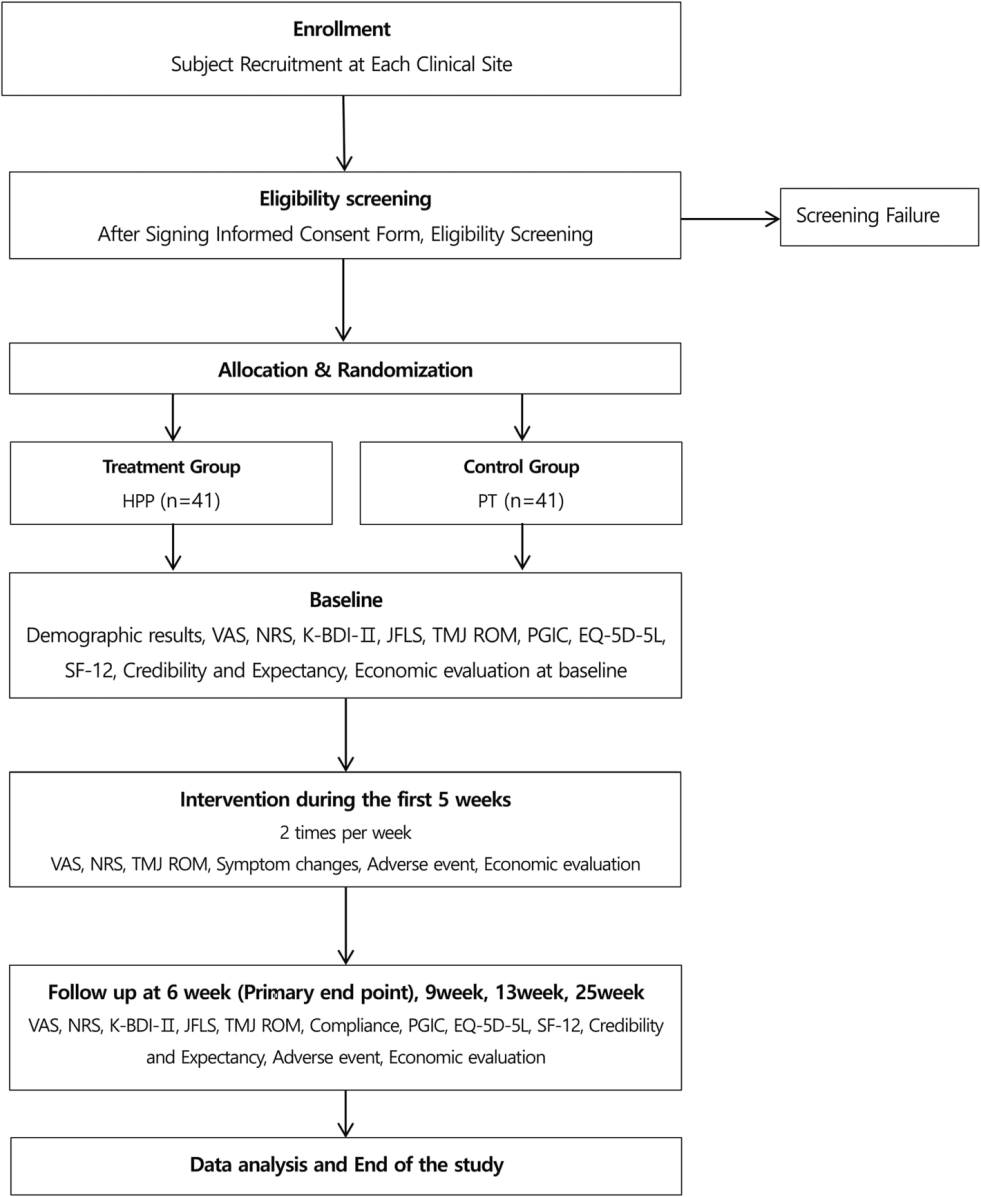
Flow chart. *EQ-5D-5 L* EuroQol-5 Dimensions 5 Levels; *JFLS* Jaw Functional Limitation Scale; *K-BDI-II* Korean-Beck Depression Inventory-II; *HPP* hominis placental pharmacopuncture; *NRS* Numeric Rating Scale; *PGIC* Patient Global Impression of Change; *PT* Physical therapy; *SF-12* Short Form Health Survey 12; *TMJ ROM* range of motion of temporomandibular joint; *VAS* Visual Analogue Scale

Participants will be recruited by physician’s recommendation, posters placed in the hospitals, and adverts placed on the hospital homepages and the metro transport system. On the first visit, a researcher will explain the study to the participants and ask them to complete and submit an informed consent form (ICF). Next, an examiner will screen the participant. Participants will be enrolled according to the inclusion/exclusion criteria, will visit 15 times, and undergo surveys either through face-to-face, telephone, or online interviews, or by distribution and return. There will be 10 treatment sessions consisting of two sessions per week for 5 weeks. Thereafter, there will be follow-up visits at 6, 9, 13, and 25 weeks from baseline. On the second visit, participants will be randomly allocated to either the HPP group or the PT group and receive the corresponding treatment. All participant management and treatment processes will be performed according to the protocol. To check compliance, we will verify whether participants adhere to the treatment schedule specified in the protocol. For the HPP group, compliance will be assessed by checking the frequency and schedule of procedures; for the PT group, compliance will be assessed by checking the frequency and schedule of TENS procedures. A study flow chart is shown in Fig. 1.

### Inclusion/exclusion criteria

#### Inclusion criteria

1. Patients with unilateral or bilateral TMJ pain
2. Patients with Visual Analogue Scale (VAS) ≥ 40 mm for the area showing TMJ pain (for patients with bilateral pain, the side with worse pain)
3. Patients complaining of persistent of sporadic TMJ pain for at least 3 months
4. Patients diagnosed as myofascial TMD (Axis I: Group 1) based on the RDC/TMD diagnostic criteria [5]
5. Patients aged 19–70 years on the date they sign the consent form
6. Patients who provide consent to participate in the trial and return the ICF

#### Exclusion criteria

1. Patients whose current pain episode developed or worsened because of a road traffic accident or traumatic injury
2. Patients diagnosed in Group 2 or 3 of Axis I based on the RDC/TMD diagnostic criteria
3. Patients who have undergone surgery related to the TMJ
4. Patients with other chronic diseases that could interfere with interpretation of the treatment effects or outcomes (e.g. rheumatoid arthritis, neoplastic disease, stroke, or myocardial infarction)
5. Patients currently taking steroids, immunosuppressants, psychiatric drugs, or other drugs that could affect the study results
6. Patients who have received HPP within the last month, or who have taken drugs that could affect pain, such as NSAIDs, within the last week
7. Pregnant or breastfeeding women
8. Patients who have finished participation in another clinical trial within the last month, who participated in another trial within 6 months of selection, or who are planning to participate in another clinical trial during the follow-up period
9. Patients with a history of hypersensitivity after HPP
10. Diabetic patients with uncontrolled blood glucose (fasting blood glucose ≥180 mg/dl)
11. Patients with aspartate aminotransferase or alanine aminotransferase at least two times the normal range at the testing centre
12. Patients with creatinine at least two times the normal range at the testing centre
13. Patients suspected to have organic disease
14. Patients with cardiac, hepatic, renal, or other serious complications
15. Patients with psychogenic disease
16. Patients who are unable to receive pharmacopuncture due to, e.g. inflammation or a wound at the relevant acupoints
17. Other situations when patients whose participation in the trial is judged by a researcher to be problematic

### Randomisation and allocation concealment

An independent statistics expert will use nQuery Advisior 7.0 (or SAS 9.0 [SAS Institute Inc., Cary, NC, USA] or SPSS 21.0 [IBM Corp., Armonk, NY, USA]) to allocate participants into each group by block randomisation in a 1:1 ratio (41 persons/group). The results of random allocation will be sealed, such that they cannot be seen from outside, and sent to each centre, where they will be managed in a double-locked container. Before treatment, a random envelope will be opened in front of the enrolled participant, the random allocation numbers assigned to each group will be recorded in an electronic chart, and no changes will be allowed after allocation. Participant enrolment at each centre will proceed by competitive enrolment.

### Blinding

Because this study does not allow the participant or treatment provider to be blinded to their group, we will use a single-blinded design, where only the assessors are blinded. Participants will be assessed away from the treatment area, by an investigator who did not participate in treatment procedures and is blinded to the treatment group; efforts will be made to prevent the investigator from knowing the allocated group. The data analyst will not be blinded to two groups. The design is open label with only the outcome assessor being blinded, so unblinding will not occur in this study.

### Interventions

At both centres, treatment will be administered by a physician who has received prior training in a standardised protocol.

#### Treatment group: HPP group

After registration, the HPP group will receive two sessions/week of HPP for 5 weeks. For HPP, we will use JHG002, prepared at the Jaseng Namyangju Herbal Dispensary. The total treatment duration, frequency, one-time dose, and site of injection for HPP were selected with reference to previous studies [11, 15–17]. A trained doctor of Korean Medicine with at least 2 years’ clinical experience will administer JHG002 pharmacopuncture with a disposable syringe (0.5 ml) directly into the designated sites, using a standardised method. The injection sites were selected based on a textbook of acupuncture and moxibustion [17], and previous studies (Additional file 2) [18, 19]. The procedure will be performed bilaterally, irrespective of the location of pain, and at each session, 0.1 ml of JHG002 will be injected per acupoint at a total of eight locations. The dose will be adjusted in the range 0.05–0.1 ml per acupoint depending on the patient’s response (i.e. pain or discomfort).

#### Control group: PT group (TENS)

The control group will receive two sessions/week of TENS for 5 weeks as a form of PT. We will use a high-frequency, low-intensity stimulus of 50–100 Hz and up to 15 mA, so that the patients feel a current but do not feel pain. At each treatment visit, a physiotherapist will administer the treatment to the bilateral TMJ for 15 minutes. Both centres will use the same TENS device—a BioTron-DX (D.M.C, Osan, South Korea).

### Concomitant treatment

Although treatments that could affect TMD will be restricted during the treatment period (weeks 1–6), in cases where severe pain makes it unavoidable, participants may receive treatment after a subinvestigator has been informed; the treatment type and frequency will be recorded in detail in the case report form (CRF). In addition, treatments unrelated to TMD will be allowed on condition that a subinvestigator is informed of the relevant facts. No restrictions will be placed on other treatments during the follow-up period (after week 6), and regardless of allocation, both groups will receive education in the causes, prevention, treatment, and management of TMD, as well as self-stretching methods [20].

### Participants schedule

The specific participant schedule is shown in Table 1.

**Table 1:** Participants schedule

	Study period										
Time point	Screening	Enrollment, Allocation	Active treatment after allocation					Follow-up			
	Week − 1	Week 0 (Baseline)	Week	Week	Week	Week	Week	Week	Week	Week	Week
			1	2	3	4	5	6	9	13	25
	Visit 1	Visit 2	Visit 2, 3	Visit 4, 5	Visit 6,7	Visit 8,9	Visit 10,11	Visit 12	Visit 13	Visit 14	Visit 15
Window period			±3	±3	±3	±3	±3	±3	±14	±14	±14
**Enrollment**											
Eligibility Screening	○	○									
Written informed consent	○										
Vital signs	○		○	○	○	○	○	○	○	○	○
Sociodemographic Characteristics, Medical History (e.g. neck pain, medication history)	○										
RDC/TMD test & analysis	○										
pattern Identification	○										
Laboratory test	○							○			
Randomised Allocation		○									
**Interventions**											
Treatment in JHG002 (experimental group)			← 2 times/week →								
Treatment in TENS (active control group)			← 2 times/week →								
**Assessments**											
Symptoms and change in medicine			○	○	○	○	○	○	○	○	○
NRS of TMJ pain/Bothersomeness			← Every visit →					○	○	○	○
VAS of TMJ pain	○		○	○	○	○	○	○	○	○	○
K-BDI-II			○					○		○	○
JFLS			○					○		○	○
TMJ range of motion (maximum mouth opening, mandibular excursive movement)	○		○	○	○	○	○	○	○	○	○
Compliance				○	○	○	○	○			
PGIC								○			○
EQ-5D-5 L			○			○		○	○	○	○
SF-12			○			○		○	○	○	○
Credibility and expectancy			○								
Adverse events			← Every visit →					○	○	○	○

*EQ-5D-5 L* EuroQol-5 Dimension-5 Level; *HPP* hominis placental pharmacopuncture, *NRS* Numeric Rating Scale; *PGIC* Patient Global Impression of Change; *RDC/TMD* Research Diagnostic Criteria for Temporomandibular Disorders; *PT* physical therapy; *SF-12* Short Form Health Survey 12; *TENS* Transcutaneous Electrical Nerve Stimulation; *TMJ* temporomandibular joint; *VAS* Visual Analogue Scale

### Dropout criteria

The trial may be stopped for a participant in the following circumstances:

1. If a severe AE is observed during the trial, or if it is believed that continuing treatment would be harmful for the participant
2. If the participant is discovered to have a disease that was not detected in testing before the trial, and that could influence interpretation of the study results
3. If the participant wants to stop participation during the trial
4. If the participant contravenes the trial plan (treatment compliance ≤ 60%)
5. If the participant has issues with completing medical procedures for TMD
6. If the participant is found to be pregnant during the trial
7. If there are any other reasons for which a subinvestigator believes that it would not be suitable for the participant to continue the trial

### Outcome measure

Outcomes will be collected at different time points during a total of 25 weeks (Table 1), and the primary end point is 6 weeks after baseline.

#### Primary outcome

##### Visual Analogue Scale (VAS) of temporomandibular pain

The primary outcome is the difference in VAS for TMJ pain between baseline and the primary end point (week 6). VAS is an assessment index in which the patient records their pain on a 100 mm line from ‘no pain’ at one end, and ‘the most severe pain imaginable’ at the other end. Patients will indicate the location on the line corresponding to their TMJ pain in the last week [21].

#### Secondary outcomes

##### Numeric Rating Scale (NRS) of TMJ pain and discomfort

The extent of TMJ pain and discomfort in the last week will be assessed using NRS. NRS is a pain scale in which the patient indicates their subjective pain as a whole number from 0 to 10. The participant is asked to report their TMJ pain and discomfort using NRS, where 0 indicates ‘no pain or discomfort’ and 10 indicates ‘the most severe pain and discomfort imaginable’ [21].

##### TMJ range of motion

A TheraBite® range of motion ruler will be used to measure the range of TMJ mouth opening and excursive movement; the measurement method will follow the guidelines provided by the International RDC/TMD Consortium [22].

##### Korean version of Beck’s Depression Index-II (K-BDI-II)

The BDI-II consists of 21 questions related to sadness, guilt, suicidal ideation, and loss of interest [23]. In this study, we will use the K-BDI-II, which has been previously demonstrated to be valid and reliable by a Korean research group [24].

Each question is scored 0–3 points, and a higher total score indicates more severe depressive tendencies. A total score of 1–10 points is ‘normal’, 10–16 points is ‘mild mood disturbance’, 17–20 points is ‘borderline clinical depression’, 20–30 points is ‘moderate depression’, 30–40 points is ‘severe depression’, and ≥ 40 points is ‘extreme depression’.

##### Jaw Functional Limitation Scale (JFLS)

The JFLS [25] assesses jaw function (mastication, mobility, and emotional and verbal expression) in the last month. The assessment consists of 20 questions, each scored 0–10, where 0 points is ‘no impairment’ and 10 points is ‘very severe impairment’. In this study, we will use the official Korean version of the JFLS, which has been previously validated [26].

##### Patient Global Impression of Change (PGIC)

The PGIC assesses overall improvements in functional limitation caused by TMD. Participants rate their improvements after treatment on a 7-point Likert scale (1 = Very much improved, 4 = No change, 7 = Very much worse). Originally developed for use in Psychology, the index is now commonly used in a variety of medical fields to assess improvements in pain [27].

##### Quality of life

We will assess quality of life using the five-level version of EuroQol-5 Dimension (EQ-5D-5 L), the EuroQol vVisual Analogue Scale (EQ-VAS), and the 12-Item Short-Form Health Survey (SF-12). The EQ-5D-5 L is the most widely used method of indirectly calculating the weights of certain health states for quality of life after a multidimensional investigation of health states. It consists of five questions about current health state (mobility, self-care, usual activities, pain, anxiety/depression), and each question is scored on a 5-point Likert scale (1 = no problems, 3 = moderate problems, 5 = severe problems). We will use the Korean version of the EQ-5D-5 L, which has been previously validated [28]. The EQ-VAS [29] is an index that assesses how good or bad a patient’s health is; the patient indicates their own health status on a 100 mm vertical line, from worst health at one end to best health at the other end. The SF-12 is a shortened version of the Short Form-36 Health Survey, which is a widely used instrument to assess health-related quality of life. The SF-12 consists of 12 questions across eight domains, and higher scores indicate a better health-related quality of life [30]. In this study, we will use the Korean version of the SF-12, which has been previously validated [31].

#### Costs data

Costs consist of medical costs, non-medical costs, and costs associated with loss of production [32]. For medical costs, we will investigate costs billed at medical institutions for medical services (official medical costs), and unofficial payments made by the individual without visiting a medical institution, such as the purchase of over-the-counter drugs, health foods, and medical devices (unofficial medical costs). We will use both micro and macro estimation methods. Non-medical costs will include travel costs, time costs, and care costs associated with the use of medical services. For travel costs, two-way transport means and costs will be estimated; for time costs, the duration of consultations at medical institutions, of two-way travel, and time spent waiting for consultations will all be included; care costs will not be separately investigated. Costs associated with loss of production comprise the economic costs due to loss of labour because of disease or early death and will be investigated using the Work Productivity and Activity Impairment Questionnaire, which is an instrument used to measure loss of production. The results of the questionnaire will be converted into costs for use in the cost-effectiveness analysis.

#### Credibility and expectancy questionnaire [33]

To measure participants’ expectations about treatment, before the start of the first treatment session participants will respond to the question, “While receiving treatment during the study period, how much do you expect your symptoms to be alleviated?” on a 9-point Likert scale (1 = not at all, 5 = somewhat, 9 = very much).

#### Safety

##### Lab tests

In order to assess participants’ suitability and detect AEs, clinical pathology tests for the following variables will be performed before the first treatment session and after the end of the treatment period (week 6): complete blood count, liver function test (including aspartate transaminase, alanine transaminase, and alkaline phosphatase), blood urea nitrogen, creatinine, erythrocyte sedimentation rate, C-reactive protein (CRP), and human chorionic gonadotropin (hCG) (only for women of fertile age). The findings will be recorded in the CRF, and if there are any abnormal results the researcher will decide whether the participant should be taken out of the trial; if necessary, further testing and therapeutic interventions will be performed.

##### Adverse events (AEs)

All AEs will be investigated at each visit and recorded in the CRF; AEs do not need to show a clear causal relationship with the relevant treatment. Serious AEs will also be recorded [34]. Since the patient’s group may be revealed during AE assessment, this will be recorded by a subinvestigator in a separate CRF. All AEs will be classified according to the three-grade classification of Spilker et al. [35]^,^ into Mild (1): not impairing the participant’s normal daily life (or function), causing minimal discomfort, and readily endured; Moderate (2): causing discomfort that interferes significantly with the participant’s normal daily life (or function); or Severe (3): making normal daily life (or function) impossible. The causal relationship of AEs with the procedure will be classified according to the World Health Organization-Uppsala Monitoring Centre (WHO-UMC) causality scale, [34] as (1) definitely related, (2) probably related, (3) possibly related, (4) probably not related, (5) definitely not related, or (6) unknown.

### Sample size

This is an investigator-initiated clinical trial, and we have calculated the required sample size with reference to similar studies and our clinical experience. First, we set a significance level of α = 0.05 (two-tailed tests), a type 2 error (β) of 0.2, and a test power of 80%. Because there are no previous studies comparing pharmacopuncture with PT for the TMJ, we selected a moderate-to-high effect size for the comparison of mean VAS between the two groups based on our clinical experience, and used Cohen’s d = 0.65 [36]. Using these parameters, we calculated the sample size using G*Power 3.1.7. Based on an effect size = 0.65, the required sample size was estimated to be 39 persons/group (total 78 persons). However, as we plan to use an analysis of covariance (ANCOVA) correcting for the outcome at baseline for our main analysis, we calculated the sample size assuming the correlation between baseline and primary endpoint to be 0.4 [(1–0.4*0.4)*78], and found that the minimum required number of participants was a total of 65 persons [37]. Thus, to account for a dropout rate of 20%, we calculated that the number of participants we need to recruit is a total of 82 persons (41 persons/group).

### Patient and public involvement statement

Patients will be involved in recruitment and conduct of the study. We will keep in touch with the patients from recruitment to 6 months after treatment. The patients will be able to contact researchers at any time to get information they need.

### Data collection and management

We will use e-CRFs via the Internet-based Clinical Research and Trial (iCReaT) management system, which is operated by the Korea Centers for Disease Control and Prevention. An investigator from the coordinating centre will distribute a standard operating procedure (SOP) to be used in trial processes such as CRF completion and data input and will provide instruction for assessors and investigators at each centre. CRF data will use double data entry, and data entry will be performed by each participating centre and the coordinating centre. After cross-checking the input data, access to the data will be blocked from all investigators except the data coordinator.

### Statistical analysis

As an RCT of chronic TMD patients, this study will compare the difference within and between groups for data on efficacy, safety, and economics. The main analysis will be the ITT analysis, and a PP analysis will also be performed. The demographic results, such as sex, age, occupation, and disease history, and the treatment expectancy will be assessed by group; continuous variables will be presented as the mean ± standard deviation (SD) and categorical variables will be presented as n (%). Differences will be tested using chi-square tests and independent *t* tests. Efficacy outcomes are the changes in continuous outcomes (VAS, NRS, K-BDI, JFLS, EQ5D-5 L, SF-12) at each time point relative to baseline. The primary outcome is the change in VAS from baseline to the primary end point (week 6) in each group. For safety outcomes, we will compare the incidence of AEs between groups; after graphing all the reported ARs in participants who have received treatment at least once, we will calculate the proportion of patients who have experienced AEs in each group, and compare these using chi-square tests and Fisher’s exact test. To compare the HPP and usual care groups, for continuous variables, we will perform an ANCOVA adjusted for the baseline outcome. For categorical variables, we will use chi-square tests of Fisher’s exact test, and to analyse within-group differences between time points, we will use paired *t* tests. Additionally, we will perform an ANCOVA adjusted for baseline outcomes and covariant factors that show a statistically significant difference between the treatment groups at baseline, and we will perform a repeated measures analysis of variance (RM ANOVA) to test differences in the changes across each visit. We will perform subgroup analyses according to RDC/TMD diagnosis (Group Ia, Ib), mouth opening, and K-BDI score. Missing values will be handled in a blinded state, mainly using multiple imputation (MI), last observation carried forward (LOCF) will additionally be used, and sensitivity analyses will be performed. All statistical analysis will be performed using SAS version 9.4 statistical package (SAS Institute, Cary, NC, USA), and statistical significance will be defined as *p* < 0.05.

### Economic evaluation

We will perform an economic analysis to investigate the cost-effectiveness of HPP compared to PT. The economic analysis can be performed from an insurer’s perspective, healthcare system perspective, and societal perspective; we have selected a societal perspective [38]. We will perform a cost-utility analysis including medical costs and non-medical costs, and costs associated with disease-related loss of production. For the final analysis results, we will present the incremental cost-effectiveness ratio (ICER) using the major economic assessment index of costs per quality-adjusted life year (QALY) gained [39]. ICER is an index that indicates the additional costs required per unit increase in effect, compared to an alternative. In addition, we will separately present the total effect (efficacy) and total costs of each treatment. For effect units, we will use QALY, which will be calculated using the quality of life derived by EQ-5D-5 L and using the area under the curve method with receiver operating characteristic (ROC) curves. For cost units we will convert all costs to 2019 South Korean Won (KRW), and apply 5% discount in accordance with the economic analysis guidelines of the Health Insurance Review and Assessment Service [40]. The treatment costs incurred by the clinical trial will be calculated by combining the treatment frequency and the unit costs; the unit costs will be calculated using the health insurance costs and the centre’s costs data. The analysis period is set to the entire clinical trial period (6 months), including the follow-up period. If estimates for the subsequent period are required, we will perform secondary analyses, such as extrapolating the costs and effects through a regression model or performing decision modelling analysis. In the baseline analysis, we will use representative values (mean, etc.) for the parameters used in the trial. To examine the uncertainty of the parameters used in the model, we will perform a sensitivity analysis; specifically, we will perform a probabilistic sensitivity analysis in which we estimate the prior distribution of the parameters, randomly extract the parameter values from these distributions, and use these results to perform a cost-effectiveness analysis.

### Data monitoring and safety monitoring

Monitoring will be performed by the monitoring coordinator at the coordinating centre. During monitoring, CRFs will be compared with evidentiary documentation, and participants’ safety data will be reviewed to verify the completeness of safety and research data. The monitoring will be conducted once every 2 months at two sites. The Trial Steering Group will meet to review conduct throughout the trial period once every 2 months. The Data Monitoring Committee was not organised as no severe AE regarding HPP or PT previously reported are expected to occur. The interim analysis will not be done in this study.

### Stopping rules

The principal investigator will decide the termination of the study; at any time during the trial period, if a participant is exposed to clear, unexpected risk or unacceptable risk, or if at least 25% of all participants experience severe AEs thought to be related to JHG002, the trial can be terminated. Severe AEs that might justify terminating the trial include local infection and severe progressive neurological deficits [41, 42].

### Victim compensation plans

In the event of direct harm related to this study, participants will receive the appropriate medical treatment as determined by a subinvestigator and will be compensated according to the terms of the trial insurance, which will be designated beforehand.

### Dissemination

The results of this trial will be registered on clinicaltrials.gov and CRIS, and will be published in an academic journal. After the publication of this article, important changes to the study protocol and any other changes will be updated regularly on the trial registration site.

## Discussion

Although HPP is used for various musculoskeletal disorders in South Korea, including TMD, there is currently a lack of evidence regarding its efficacy for TMD. In this study, we will conduct an RCT to assess the effectiveness, safety, and economics of HPP in chronic TMD patients compared with PT.

Pharmaco-acupuncture is a form of treatment used in complementary and alternative medicine that combines acupuncture with herbal medicine. By injecting purified herbal medicine extracts into acupoints it provides a combination of the chemical effect from herbal medicine and the physical stimulation effect of needling. Because the herbal extracts are absorbed directly into the treatment site without passing through the digestive system, the effects can be observed immediately; the treatment can be given to patients who cannot receive oral administration and the dose can be easily adjusted [17]. In South Korea, the procedure is performed by doctors of Korean medicine, who are experts in acupuncture and herbal medicine prescriptions.

In this study, we will use HPP as the pharmacopuncture formulation. Hominis placenta (HP), a human placental extract, is the main component of HPP, and has been used in traditional Korean medicine to treat chronic diseases, including frailty, cough, anorexia, and fatigue, by enhancing the body’s resistance. HP is known to be effective for liver regeneration, regulation of the endocrine system [43], antioxidation, nerve regeneration [44], immune enhancement, regulation of menopausal syndrome [45] and wound healing [46]. According to a systematic review [47], HPP has been demonstrated to have anti-inflammatory and antioxidative effects in in vitro and in vivo studies, while clinical trials have reported that HPP is effective for musculoskeletal and neurological conditions, such as alleviating pain, measured by VAS, in chronic ankle sprain patients [48], improving restricted shoulder motion in patients with complex regional pain syndrome [49], and ameliorating peripheral facial nerve palsy [50]. Although HPP is also used in TMD, most studies are case reports. Concomitant treatments make it impossible to identify the effects of HPP alone, although there have been few reports of adverse effects. Therefore, there is a need to collect evidence for HPP through well-designed RCTs.

The advantages of this study are as follows. First, the patient group selection process is clear. In previous studies [51–53], the TMD diagnostic criteria were ambiguous, or there was no distinction between acute and chronic disease, which made it difficult to ensure homogeneity of the patient group. Our study uses a validated Korean version of the RDC/TMD diagnostic criteria, which are standardised and the most widely used criteria [26] and we will only enrol chronic patients, in order to form a precisely defined patient group. Second, the selection of intervention for the treatment group is appropriate. We decided to treat chronic TMD using HP, which is known to aid recovery of injured tissue because it contains growth factor, which promotes wound healing [54]. Moreover, pharmacopuncture is the method of injecting herbal medicine extracts into acupoints; because herbal medicines contain a combination of several ingredients, it is difficult to precisely test the efficacy and safety of individual ingredients, and it is difficult to standardise injected formulations. In anticipation of this debate, we chose HPP, which uses a single active ingredient, is stable, and can be considered relatively safe, since the same extract is already used as an injectant (Laennec®) [50]. Third, the selection of intervention for the control group is objective. According to guidelines for TMD treatment [7], over 90% of TMD can be controlled by conservative treatment, such as PT or drug therapy, but evidence of the effects of several types of conservative treatment is unclear. In a Cochrane review of drug therapy for TMD, [55] it was reported that, even though drug therapy, NSAIDs, benzodiazepines, antispasmodics, and muscle relaxants can be used, there is a lack of clear evidence for their effectiveness. Therefore, considering the statistical data reflecting the clinical reality in guidelines [56, 57], we selected PT (TENS) for the control group.

Nevertheless, our study also has some limitations. First, the study design makes it impossible to blind participants and physicians. However, the physicians and assessors were separated; the assessors did not participate in treatment procedures, performed assessments in a separate space, and were thoroughly blinded to group allocation. Second, psychiatric and psychological factors are known to be important predictive factors for therapeutic effect in TMD [58], but we will not match these factors in the screening stage. This is to minimise selection bias, and, to minimise the effects of these psychiatric and psychological factors, group allocation will be randomised, and we will perform subgroup analysis by K-BDI score to investigate the effects of these factors. Third, because this study includes long-term follow-up, it could be difficult to maintain compliance during follow-up. Accordingly, we will make efforts to improve compliance, such as regular phone calls to participants.

We hope the data from this study will contribute to evidentiary data for standard Korean medicine clinical treatment guidelines. In addition, the study design and methodology can be used as a reference for future clinical trials of acute and subacute TMD or other musculoskeletal disease, as well as studies of other types of pharmacopuncture.

### Trial status

The current protocol is version 2.3 (2 February 2020). First participant was enrolled on 17 October, 2019. We anticipate that the recruitment will be completed by October 2020.

## Supplementary information

**Additional file 1.** SPIRIT checklist.

**Additional file 2.** Acupoint locations and standards used for locating acupoints used in this trial.

**Additional file 3.** Trial committee organisation and roles.

## Data Availability

The datasets analysed during the current study are available from the corresponding author on reasonable request.

## Abbreviations

AAOP: American Academy of Orofacial Pain
AE: Adverse event
ANCOVA: Analysis of covariance
CRF: Case report form
CRP: C-reactive protein
EQ-5D-5 L: EuroQol-5 dimensions 5 levels
EQ-VAS: EuroQol Visual Analogue Scale
hCG: Human chorionic gonadotropin
HIRA: Health Insurance Review and Assessment Service
HP: Hominis placenta
HPP: Hominis placental pharmacopuncture
ICER: Incremental cost-effectiveness ratio
ICF: Informed consent form
iCReaT: Internet-based clinical research and trial
IRB: Institutional review board
ITT: Intention-to-treat
JFLS: Jaw Functional Limitation Scale
K-BDI: Korean version of Beck’s Depression Index
KHIDI: Korea Health Industry Development Institute
LOCF: Last observation carried forward
MI: Multiple imputation
NRS: Numeric Rating Scale
NSAIDs: Non-steroidal anti-inflammatory drugs
PA: Pharmaco-acupuncture
PGIC: Patient global impression of change
PP: Per protocol
PT: Physical therapy
QALY: Quality-adjusted life year
RCT: Randomised control trial
RDC/TMD: Research diagnostic criteria for temporomandibular disorders
RM ANOVA: Repeated measures analysis of variance
ROC: Receiver operating characteristic
ROM: Range of motion
SD: Standard deviation
SF-12: 12-Item Short-Form Health Survey
SOP: Standard operating procedure
SSRI: Selective serotonin reuptake inhibitor
TENS: Transcutaneous electrical nerve stimulation
TMD: Temporomandibular disorder
TMJ: Temporomandibular joint
VAS: Visual Analogue Scale
WHO-UMC: World Health Organization-Uppsala Monitoring Centre
