# Supplementary material for: Efficacy, safety, and economic assessment of hominis placental pharmacopuncture for chronic temporomandibular disorder: a protocol for a multicentre randomised controlled trial
Source: Trials. 2020 Jun 15;21:525. doi: 10.1186/s13063-020-04442-8 (PMC7294621; doi:10.1186/s13063-020-04442-8)
Supplement: Supplementary file 2 — Additional file 2. Acupoint locations and standards used for locating acupoints used in this trial. [file 13063_2020_4442_MOESM2_ESM.docx]

Additional file 2. Acupoint locations and standards used for locating acupoints used in this trial

| .**Acupoints** | **Procedures for locating the acupoints** |
| --- | --- |
| SI19 (聽宮) | Between the targus and the mandibular joint where a depression is formed when the mouth is slightly open. |
| GB20 (風池) | In the anterior region of the neck, inferior to the occipital bone, in the depression between the origins of sternocleidomastoid and the trapezius muscles. |
| GB21 (肩井) | In the posterior region of the neck, at the midpoint of the line connecting the spinous process of the seventh cervical vertebra (C7) with the lateral end of the acromion. |
| TE17 (翳風) | In the anterior region of the neck, posterior to the ear lobe, in the depression anterior to the inferior end of the mastoid process. |
| ST7 (下關) | On the face, in the depression between the midpoint of the inferior border of the zygomatic arch and the mandibular notch. |
| ST6 (頰車) | On the face, one fingerbreadth (middle finger) anterosuperior to the angle of the mandible. |
| LI18 (扶突) | On the anterior aspect of the neck, at the same level as the superior border of the thyroid cartilage, between the anterior and posterior borders of the sternocleidomastoid muscle. |
| EX-HN5 (太陽) | In the region of the temples, in the depression about one finger-breadth posterior to the midpoint between the lateral end of the eyebrow and the outer canthus. |
