# Supplementary material for: Efficacy, safety, and economic assessment of hominis placental pharmacopuncture for chronic temporomandibular disorder: a protocol for a multicentre randomised controlled trial
Source: Trials. 2020 Jun 15;21:525. doi: 10.1186/s13063-020-04442-8 (PMC7294621; doi:10.1186/s13063-020-04442-8)
Supplement: Supplementary file 3 — Additional file 3. Trial committee organisation and roles. [file 13063_2020_4442_MOESM3_ESM.docx]

**Additional file 3. Trial committee organization and roles**

**Principal investigator (PI) and research physicians**

**Organization:** Jaseng medical foundation

**Organization of Steering committee and member appointment**: In-Hyuk Ha, Yoon Jae Lee, Kyoung Sun Park, and Jongho Kim

**Roles:** Communication and exchange of opinion with PI at each site (Meeting once every week before IND approval /once every month during recruiting)

Preparation of IRB documents and CRF

IND submission and approval

Trial management (randomized allocation management, AE data collection at each site, participant enrollment supervision, study site inspection and visits, budget allocation and management)

Data collection, quality control, monitoring, and analysis

**Trial steering committee (TSC)**

**Organization and role:** All authors of this manuscript

**Contributions:** Protocol revision and decision on final protocol (meeting once every two months)

Organization of Trial Management Committee and member appointment

Designation of participant recruitment study sites

Inspection of study progress, and decision on protocol revision, if needed

Determination of study result publication timing and method

Decision on authorship in accordance with Authorship eligibility guidelines

**Trial management committee**

**Organization:** PI and investigators at each clinical trial participant enrollment site

**Organization and role:** Submission and obtaining study protocol approval from relevant IRB of each study site

Clinical trial execution following protocol (e.g. participant recruitment, enrollment, data collection, CRF entry)

Collection and report of AEs
